# Advanced Platelet-Rich Fibrin Plus Sealed Exclusively with Glass Ionomer Cement: Setting a New Standard for Healing, Aesthetics and Predictive Modelling in Regenerative Endodontics

**DOI:** 10.3390/ma18184421

**Published:** 2025-09-22

**Authors:** Dubravka Turjanski, Dragutin Lisjak, Petra Bučević Sojčić, Jelena Valpotić, Tea Borojević Renić, Kristina Goršeta, Domagoj Glavina

**Affiliations:** 1 Department of Paediatric and Preventive Dentistry, Dental Polyclinic Zagreb, Perkovčeva 3, 10000 Zagreb, Croatia; jelena_strazanac@yahoo.com; 2 Faculty of Mechanical Engineering and Naval Architecture, University of Zagreb, Ivana Lučića 5, 10000 Zagreb, Croatia; dragutin.lisjak@fsb.unizg.hr; 3 Department of Paediatric and Preventive Dentistry, School of Dental Medicine, University of Zagreb, Gundulićeva 5, 10000 Zagreb, Croatia; pbucevic@sfzg.unizg.hr (P.B.S.); gorseta@sfzg.unizg.hr (K.G.); glavina@sfzg.unizg.hr (D.G.); 4 Department of Prosthodontics, Dental Polyclinic Zagreb, Perkovčeva 3, 10000 Zagreb, Croatia; tea5bor@yahoo.com

**Keywords:** regenerative endodontics, advanced platelet-rich fibrin plus (A-PRF+), glass ionomer cement (GIC), calcium hydroxide apexification, root maturation, pulp vitality, laser Doppler flowmetry (LDF), apical closure, biomaterials, tooth discolouration

## Abstract

Regenerative endodontic approaches for immature necrotic permanent teeth must balance biological efficacy, clinical practicality and long-term aesthetic outcomes. This study evaluates a novel regenerative protocol using autologous advanced platelet-rich fibrin plus (A-PRF+) scaffold sealed exclusively with glass ionomer cement (GIC) and compares it to conventional calcium hydroxide apexification used as the control. Twenty-eight patients were prospectively enrolled and followed for 12 months alongside a retrospectively selected historical control group. Outcomes were evaluated through standardised blinded clinical, radiographic and vitality assessments. The A-PRF+ protocol demonstrated significantly faster periapical healing, superior root lengthening, increased dentinal wall thickness and apical closure (*p* < 0.0001), with excellent aesthetic outcomes and no reported tooth discolouration. Pulpal blood flow measured by laser Doppler flowmetry indicated vitality restoration in 93% of cases. Preliminary linear regression identified treatment duration as a significant predictor of apical closure (*p* < 0.0001), with possible enhancement by additional patient-specific variables. These findings validate the A-PRF+ protocol as a highly effective, aesthetically favourable and predictable regenerative strategy, establishing a new benchmark for the management of immature necrotic teeth and laying the foundation for personalised predictive endodontic care. Future studies should include multicentre randomised controlled trials to confirm long-term clinical sustainability and generalisability.

## 1. Introduction

The management of non-vital immature permanent teeth, typically resulting from dental trauma or caries, presents significant challenges in paediatric dentistry due to incomplete root development, thin dentinal walls and compromised structural integrity [1,2]. Without appropriate intervention, these teeth carry an increased risk of loss, which negatively impacts functional stability, aesthetics and patient quality of life [3]. Treatment strategies should therefore prioritise tooth preservation, continued root maturation and reinforcement of the root structure prior to definitive restorative procedures [4,5,6].

Traditional apexification methods using calcium hydroxide or calcium silicate–based cements, such as mineral trioxide aggregate (MTA) and Biodentine, are well established for promoting apical healing and barrier formation [7]. However, calcium hydroxide apexification typically requires a prolonged treatment duration, increasing the risks of reinfection and susceptibility to root fracture due to reduced dentinal strength. MTA, although biocompatible and capable of effective sealing, has disadvantages including tooth discolouration, extended setting times and handling difficulties. Biodentine, while easier to handle and faster setting, may still cause discolouration and its cost limits routine use [8,9,10].

Regenerative endodontic procedures (REPs) have emerged as biologically based alternatives, aiming not only to eliminate infection but also to promote root development, increased dentinal wall thickness and apical closure through stem cell activation, growth factors and scaffolds. Early strategies used intracanal blood clots, which allowed partial regeneration but limited revascularisation [11]. Contemporary approaches incorporate improved disinfection, antibiotic pastes, standardised irrigation and induction of apical bleeding [12]. The current REP guidelines from the American Association of Endodontists (AAE) and European Society of Endodontology (ESE) emphasise minimal instrumentation, effective disinfection and use of intracanal scaffolds with biocompatible sealing materials. Success is assessed by primary outcomes of symptom resolution and periapical healing, secondary outcomes of root lengthening, dentinal wall thickening and apical closure and tertiary outcomes such as pulp vitality restoration. Additional indicators include pain relief, absence of inflammation, radiographic repair, sensibility responses, patient satisfaction and prevention of tooth discolouration [13].

Among scaffolds, leukocyte- and platelet-rich fibrin (L-PRF) stands out due to its autologous origin, biocompatibility and sustained release of growth factors that support tissue regeneration [14,15]. Recent studies have confirmed that platelet concentrates, such as platelet-rich plasma and platelet-rich fibrin, promote more predictable root development, enhanced periapical healing and higher rates of pulp vitality than blood clots [4,10,14,16,17,18,19]. Advanced platelet-rich fibrin plus (A-PRF+), produced through low-speed centrifugation, contains a higher concentration of cells and facilitates prolonged growth factor delivery. Clinical evidence has demonstrated its superior regenerative capacity in immature necrotic teeth [20,21,22]. However, scaffold placement and coronal sealing remain clinical challenges in regenerative endodontics. 

Compared to MTA and Biodentine, glass ionomer cements (GICs) offer practical benefits in REPs, including lower cost, ease of use and pre-encapsulated forms that streamline application [23]. GICs chemically bond to dentine, seal against microleakage [24] and release fluoride, aiding caries prevention [25,26]. They are biocompatible, non-toxic and do not cause discolouration—overcoming a key drawback of MTA [8,9,27,28]. Though mechanically weaker than Biodentine [29], GICs are suitable for intra-orifice barriers and cavity sealing without hindering regeneration. Their role as the sole coronal barrier, however, remains underexplored.

A core limitation of current REP protocols is confinement of scaffolds to the root canal, excluding the pulp chamber. This hinders full reconstruction of the pulp–dentine complex and may limit uniform revascularisation and reinnervation. To address this, a novel REP protocol has been developed using A-PRF+ as the sole scaffold, extending from apex to coronal cavity, sealed directly with GIC, omitting MTA or Biodentine as intermediate barriers. This strategy combines the biological benefits of A-PRF+ with the clinical efficiency and aesthetic stability of GIC.

Over a 12-month observation period, the regenerative efficacy of this A-PRF+ protocol will be evaluated according to the following objectives:

- To assess clinical outcomes, including symptom resolution and treatment success rates;
- To quantify radiographic changes in root length, dentinal wall thickness and apical diameter;
- To confirm revascularisation and reinnervation via laser Doppler flowmetry and pulp vitality testing.

It is hypothesised that this protocol will outperform conventional calcium hydroxide apexification, achieving reliable apical closure, progressive root development and restoration of physiological pulp vitality. The importance of this research lies in overcoming two major shortcomings of current regenerative practice: the lack of scaffold extension into the coronal chamber and the absence of evidence for GIC as the exclusive coronal seal. By directly addressing these gaps, this protocol aims to establish a simplified, biologically effective and aesthetically favourable approach for managing immature necrotic permanent teeth. The expected outcome is the formation of a fully mature root with continuous canal and coronal spaces, free from calcific barriers, accompanied by restitution of the pulp chamber roof and regeneration of vascularised and innervated pulp-like tissue. Radiographically, such a tooth would appear virtually indistinguishable from a fully developed permanent tooth, aside from the presence of restorative material.

## 2. Materials and Methods

### 2.1. Study Design

The present investigation was designed as a prospective interventional clinical study employing a historical control group. Patients presenting to the Department of Paediatric and Preventive Dentistry, University Hospital Centre Zagreb, Croatia, with necrotic, immature, permanent single-rooted teeth were prospectively enrolled and treated using the A-PRF+ revascularisation protocol. The control group comprised an equal number of patients who had previously undergone traditional calcium hydroxide apexification at the same institution.

The study was conducted in accordance with the Declaration of Helsinki and approved by the Ethics Committee of the School of Dental Medicine, University of Zagreb (No. 05-PA-24-2/2018). Retrospective registration was completed at ClinicalTrials.gov (NCT07092488; date of registration: 28 July 2025), in accordance with the EU Clinical Trials Regulation 536/2014 (effective 31 January 2022).

The treatment protocol was explained to all patients and their legal guardians both verbally and in writing, and written informed consent was obtained prior to enrolment.

Patients were recruited consecutively over a period of approximately two years, beginning in March 2018, following ethical approval. Each patient was followed for 12 months from the initiation of treatment. Two cases were excluded due to irregular attendance at follow-up appointments, resulting in 28 evaluable cases in the experimental group. Records of single-rooted teeth treated with calcium hydroxide apexification during the same period were screened for eligibility in the control group. Of 47 eligible cases, 28 were randomly selected to match the experimental cohort.

### 2.2. Sample Size

Sample size estimation was based on the means and standard deviations of root lengthening (RL), dentinal wall thickening (DWT) and apical closure (AC) measured at the 6-month follow-up in the A-PRF+ group, as well as on corresponding parameters obtained at the end of treatment in 10 randomly selected calcium hydroxide apexification controls (Table 1). The largest required sample size, calculated for RL, was used to determine the overall study size.

To achieve 95% statistical power at a significance level of 0.05, a minimum of 25 teeth per group was required, accounting for an anticipated 20% loss to follow-up. In total, 28 teeth were included in each group over the course of the study period.

Sample size calculations were performed using the MathWorks® Statistics and Machine Learning Toolbox, version R2023b (MathWorks Inc., Natick, MA, USA). Post hoc recalculations incorporating data from the 9- and 12-month follow-ups confirmed that the initial estimate was sufficient and adequately powered.

### 2.3. Participants and Eligibility Criteria

#### 2.3.1. Experimental Group (A-PRF+ Regenerative Procedure)

Eligible participants in the experimental group were systemically healthy patients (ASA classification I or II), aged 8–18 years, presenting with immature permanent single-rooted teeth exhibiting pulp necrosis due to caries or dental trauma. Inclusion criteria required a preserved tooth crown, negative responses to cold and electric pulp tests and radiographic evidence of incomplete root development according to the Cvek classification stages I–IV (Stage I: root length less than half completed; Stage II: root length half completed; Stage III: root length two-thirds completed; Stage IV: wide-open apical foramen with nearly complete root formation). An apical diameter of ≥0.5 mm was also required. Patient selection followed the guidelines established by Wei et al. [30].

Exclusion criteria were systemic disease, known allergy to treatment components, complicated crown or root fractures, radiographic signs of ankylosis or root resorption, ongoing orthodontic treatment or inability to comply with follow-up protocols (e.g., needle phobia, poor cooperation). Patients were also excluded if the treated tooth sustained trauma during follow-up or if scheduled recalls were missed.

To minimise selection bias, eligible patients were consecutively enrolled throughout the study period.

#### 2.3.2. Control Group (Calcium Hydroxide Apexification)

The control group comprised patients aged 8–18 years with immature permanent single-rooted teeth diagnosed with pulp necrosis due to caries or trauma, who had previously undergone calcium hydroxide apexification at the same institution. Inclusion criteria were a preserved crown, complete clinical documentation and at least two diagnostic radiographs (preoperative and final recall). Radiographic evidence of incomplete root development (Cvek stages I–IV) and an apical diameter of ≥0.5 mm were mandatory.

Radiographic comparability was confirmed by measuring mesio-distal and inciso-cervical crown dimensions; image pairs with dimensional discrepancies greater than 10% were excluded, following established best practices using standardised paralleling techniques [31].

Further exclusion criteria included treatment durations less than 6 months or greater than 12 months, incomplete documentation or inconsistencies in recorded protocols. To reduce selection bias, 28 eligible cases were randomly selected from the historical cohort.

### 2.4. A-PRF+ Revascularisation Protocol (Experimental Group)

All procedures were performed by experienced paediatric dental specialists at the Department of Paediatric and Preventive Dentistry, University Hospital Centre Zagreb, Croatia, under strict aseptic conditions, including the use of sterile gloves, drapes, rubber dam isolation and thorough tooth surface disinfection with 5% sodium hypochlorite.

#### 2.4.1. First Appointment

Following local anaesthesia (Mepivastesin, 3M ESPE, Seefeld, Germany) and rubber dam isolation (Elastidam, Coltene, Altstätten, Switzerland), an access cavity was prepared. Working length was established using an electronic apex locator (Endometer ES-02; Artronic d.o.o., Zagreb, Croatia), set 0.1 mm short of the radiographic apex.

Minimal canal instrumentation was performed using a size 25 H-file (Medin a.s., Nové Město na Moravě, Czech Republic). The canal was irrigated with 10 mL of 5% sodium hypochlorite (Gram-Mol d.o.o., Zagreb, Croatia) via a side-vented needle, followed by irrigation with 10 mL of sterile saline. After drying with sterile paper points (Diadent MMPP; DiaDent Group International, Cheongju, South Korea), calcium hydroxide paste (Calcicur; Voco GmbH, Cuxhaven, Germany) was placed using a syringe. A sterile cotton pellet was inserted, and the cavity was sealed with encapsulated glass ionomer cement (GC Fuji IX GP Fast; GC Corporation, Tokyo, Japan).

#### 2.4.2. Second Appointment (Two Weeks After Initial Treatment)

The access cavity was reopened. Minimal mechanical instrumentation was repeated, followed by irrigation with 10 mL of 5% sodium hypochlorite and 10 mL of sterile saline. The canal was dried with sterile paper points.

Triple antibiotic paste (TAP) was prepared by triturating ciprofloxacin (Cipromed, 250 mg tablets; Pliva, Zagreb, Croatia), metronidazole (Medazol, 400 mg tablets; Belupo, Koprivnica, Croatia) and minocycline (Hiramicin, 100 mg tablets; Pliva, Zagreb, Croatia) into fine powders. Equal amounts were weighed to the nearest 10 mg, mixed in a 1:1:1 ratio and diluted with sterile saline to achieve a final concentration of approximately 5 mg/mL, in line with regenerative endodontic recommendations [30].

TAP was placed below the cementoenamel junction using a size 25 lentulo spiral (Dentsply Maillefer, Ballaigues, Switzerland) to minimise the risk of discolouration from minocycline. The cavity was sealed with a sterile cotton pellet and glass ionomer cement.

#### 2.4.3. Third Appointment (Three Weeks After Initial Treatment)

Immediately prior to the procedure, autologous A-PRF+ was prepared via venepuncture of peripheral blood into anticoagulant-free sterile tubes (VACUETTE® Z Serum Clot Activator; Greiner Bio-One, Kremsmünster, Austria), followed by centrifugation (PRF DUO; Process for PRF, Nice, France) at 1300 rpm for 8 minutes. This yielded three fractions: red blood cells (bottom layer), A-PRF+ clot (middle) and platelet-poor plasma (PPP; top). The PPP was aspirated and reserved for irrigation.

The A-PRF+ clot was separated from the red cell layer using sterile scissors, then divided—part for membrane formation and part fragmented for intracanal placement.

Under local anaesthesia without vasoconstrictor (Mepivastesin, 3M ESPE) and rubber dam isolation, the access cavity was re-entered. The tooth and cavity were disinfected with 5% sodium hypochlorite. TAP was removed with 10 mL of sterile saline, followed by a final rinse using the reserved PPP.

Bleeding was induced by over-instrumentation of the periapical tissues using a size 25 H-file. The canal was filled with A-PRF+ fragments using a plugger, and an A-PRF+ membrane was placed coronally.

The cavity was permanently restored with glass ionomer cement and thermocured for 60 s using an LED curing light (Bluephase G2; Ivoclar Vivadent, Schaan, Liechtenstein) at 1200 mW/cm^2^. Final restoration was completed using encapsulated GIC according to clinical protocol.

### 2.5. Calcium Hydroxide Apexification Therapy (Control Group)

Control group patients had previously undergone calcium hydroxide apexification. Following local anaesthesia and rubber dam isolation, an access cavity was prepared. Minimal mechanical instrumentation of the root canal was performed.

The canal was irrigated with 10 mL of 1% sodium hypochlorite, followed by 10 mL of sterile saline and dried with paper points. Calcium hydroxide paste was placed as an intracanal medicament. The cavity was temporarily sealed with a sterile cotton pellet and glass ionomer cement.

Calcium hydroxide dressings were renewed every 1–3 months according to clinical and radiographic assessments. Minor variations in paste type or irrigant used were allowed, reflecting standard clinical practice at the time. All procedures were carried out by paediatric dental specialists, and records were carefully reviewed for eligibility.

The endpoint of apexification therapy was defined as the point at which a radiographic apical barrier was evident, allowing conventional obturation. In cases where treatment was altered, the endpoint included apical plug placement using MTA or transition to another regenerative or apexification approach [32]. Criteria followed those outlined by Murray [33].

### 2.6. Follow-Up and Evaluation Methods

#### 2.6.1. Recall Schedule

Follow-up was scheduled at 1, 3, 6, 9 and 12 months post-intervention. These intervals were chosen to ensure adequate monitoring of root development.

Each visit included a clinical examination, pulp vitality assessment and standardised radiography. All evaluations were performed by independent clinicians who had not been involved in the initial treatment, in order to minimise examiner bias. Baseline radiographs were those taken immediately post-intervention.

#### 2.6.2. Clinical Examination

Each follow-up involved standardised inspection for discolouration (by comparison with the contralateral tooth) and clinical signs of inflammation (e.g., redness, swelling or sinus tract formation).

Palpation assessed tenderness or swelling, and percussion evaluated the response to tapping. Abnormal mobility or other pathological signs were recorded.

For the control group, available clinical documentation was reviewed and compared with these parameters; however, retrospective records might not have comprehensively captured all signs and symptoms.

#### 2.6.3. Clinical Evaluation of Restorations

Restorations were assessed at each follow-up visit by experienced clinicians using established clinical criteria adapted from the modified United States Public Health Service (USPHS) system [34]. Parameters evaluated included retention, marginal discolouration, anatomical form, marginal adaptation, the presence of secondary caries and surface texture.

The purpose of this evaluation was to monitor the clinical performance of glass ionomer cement (GIC) as the final restorative material over time. Assessments were qualitative and were not formally scored or subjected to statistical analysis.

#### 2.6.4. Pulp Vitality Testing

Pulp sensibility was assessed at each follow-up appointment using cold spray (Plurasol Kältespray; Pluradent GmbH, Dresden, Germany) and electric pulp testing (PARKELL Model PT-20; Parkell Inc., Edgewood, NY, USA), in accordance with the manufacturers’ instructions. Both tests were applied to the treated and contralateral teeth, and responses were documented as positive or negative.

Laser Doppler flowmetry (LDF) was performed once per patient, at either the 9- or 12-month recall, to quantitatively assess pulpal blood flow as an objective indicator of vitality [35]. Measurements were obtained using a Laser Blood Flow Monitor MBF3/D (Moor Instruments Ltd., Axminster, UK), operating at a wavelength of approximately 780–785 nm, 1.0 mW output power and a sampling frequency of approximately 40 Hz. Only one channel was used, and the flux parameter was recorded over a two-minute period.

To ensure consistent probe positioning and minimise soft tissue interference, a custom holder was fabricated for each patient using Kerr Pentron Correct Plus (Kerr UK Ltd., Uxbridge, UK) hydrophilic impression material. The holder secured the probe at the cervical third of the crown, at a distance of approximately 5 mm from the gingival margin [36].

A continuous flux signal was interpreted as a positive response (+), while the absence or discontinuity of the signal was recorded as negative (−). All vitality assessments were performed by clinicians blinded to treatment allocation, in order to minimise examiner bias.

#### 2.6.5. Radiographic Analysis

Standardised periapical radiographs were obtained at all follow-up appointments. In the A-PRF+ group, radiographs were taken at baseline and at 1, 3, 6, 9 and 12 months using a film holder to ensure consistent angulation and minimise distortion. For the control group, only baseline and final radiographs were available, and these were retrospectively assessed for distortion by comparing mesio-distal and inciso-cervical crown dimensions; images with discrepancies greater than 10% were excluded in line with best practice paralleling techniques [31].

Measurements were performed using Scanora 5.1 software (Soredex, build 5.1.0.9; IAM version 4.05.0009, Tuusula, Finland). Region of Interest (ROI) tools were used for analysis: the ROI Measuring Length tool enabled precise linear measurements, the ROI Equalizer tool adjusted contrast to enhance anatomical boundaries and the ROI Magnification tool provided zoom for improved measurement accuracy. Root length was measured as the linear distance from the cementoenamel junction (CEJ) to the apex, with mesial and distal values averaged to obtain the mean root length. Dentinal wall thickness was calculated at the coronal two-thirds of the baseline root length by subtracting the pulp space width from the total root width at that level, then halving the result to obtain the mean dentinal wall thickness. Apical diameter was measured as the horizontal width of the apical foramen. Each measurement was recorded as a single value per tooth.

This protocol followed the methodology of Saoud et al. [37] to ensure consistency with established radiographic assessments in immature necrotic teeth.

All measurements were performed independently by two trained examiners, who were calibrated using standardised training cases until measurement variation was ≤0.1 mm—the minimum detectable value of the software. Radiographs were randomly coded to blind examiners to both case identity and follow-up time point. Any discrepancies exceeding the acceptable threshold were resolved by joint review and consensus. Although formal inter-examiner reliability statistics were not calculated, this protocol ensured reproducible and consistent measurement accuracy.

To quantify treatment effects, changes in root length (root lengthening, RL), dentinal wall thickness (dentinal wall thickening, DWT) and apical diameter (apical closure, AC) were calculated as follows:

- Root lengthening (RL): difference between follow-up and baseline mean root lengths;
- Dentinal wall thickening (DWT): difference between follow-up and baseline mean dentinal wall thickness;
- Apical closure (AC): difference between baseline and follow-up apical diameters (baseline minus follow-up).

By focusing on dimensional change rather than absolute values, anatomical variation was minimised and comparability between cohorts was improved. All changes were reported as mean ± SD (mm). Periapical lesions were noted as present (+) or absent (−) by the same examiners.

#### 2.6.6. Outcome Measures

The primary outcomes of this study were quantitative changes in root length (root lengthening, RL), dentinal wall thickness (dentinal wall thickening, DWT) and apical diameter (apical closure, AC), each calculated as the difference between baseline and follow-up radiographic measurements. All values were reported as mean ± standard deviation (SD) in millimetres (mm).

Secondary outcomes included the presence or absence of periapical pathology, pulp vitality responses (assessed by cold pulp testing, electric pulp testing and laser Doppler flowmetry), tooth survival and the incidence of adverse clinical findings such as discolouration or pathological mobility.

Categorical variables—including vitality responses and clinical symptoms—were recorded as present (+) or absent (−), with results expressed as percentages.

#### 2.6.7. Statistical Analysis

Statistical analysis was performed using the Statistics and Machine Learning Toolbox, version R2023b (MathWorks Inc., Natick, MA, USA). Descriptive statistics were used to summarise all variables.

Group comparisons at baseline were conducted using Student’s *t*-test for continuous, normally distributed data; Mann–Whitney *U* test for non-normally distributed data; and either Fisher’s exact test or chi-square test for categorical variables.

Normality of residuals was assessed using both the Shapiro–Wilk and Kolmogorov–Smirnov tests. Parametric data were compared using analysis of variance (*ANOVA*), while non-parametric data were analysed using the Kruskal–Wallis *H* test. A *p* value < 0.05 was considered statistically significant.

Linear regression analysis was conducted to develop a predictive model for apical closure in the A-PRF+ group. Categorical variables, such as clinical symptoms and pulp vitality responses, were reported as counts and percentages and compared using the chi-square test or Fisher’s exact test.

#### 2.6.8. Allocation and Blinding

This was an open-label study in which participants, clinicians and outcome assessors were not masked during treatment. However, measures were implemented to minimise bias.

Allocation to groups was non-randomised by necessity: the experimental group was prospectively enrolled, while the control group was selected retrospectively from eligible historical cases. To reduce selection bias, 28 control cases were randomly chosen from a pool of 47 eligible records, as detailed in Section 2.1.

Blinding was applied to radiographic assessment: all radiographs were randomly coded to conceal case identity and follow-up time point. Two trained examiners, who were not involved in treatment delivery, performed all linear measurements independently. Any discrepancies beyond the acceptable threshold were resolved by joint review and consensus.

Follow-up examinations—including clinical evaluations and pulp vitality testing—were conducted by independent clinicians who had not participated in the initial treatment phase. These measures served to minimise examiner bias and improve the internal validity of outcome assessments.

## 3. Results

### 3.1. Initial Study Population Profile

At baseline, the A-PRF+ and control groups were comparable in terms of age, sex, aetiology, tooth type and the prevalence of periapical lesions, with no statistically significant differences detected between groups (Student’s *t*-test for age; Fisher’s exact test for sex, aetiology, tooth type and periapical lesions). Although the mean apical diameter was higher in the A-PRF+ group (1.63 ± 0.90 mm) than in the control group (1.14 ± 0.44 mm), this difference did not reach statistical significance according to the Mann–Whitney *U* test (*p* = 0.08). A summary of these baseline characteristics is provided in Table 2.

### 3.2. Clinical Outcomes

All 28 teeth in the experimental group (A-PRF+ revascularisation) completed the scheduled 12-month prospective follow-up, with no losses to follow-up and no cases of tooth loss during the observation period. In the control group (retrospective calcium hydroxide apexification), 28 cases were randomly selected, with a mean treatment duration of 8.6 ± 2.8 months (range 6–12 months), and no tooth loss was reported.

No clinical symptoms—such as tenderness to percussion or palpation, swelling, sinus tract formation or spontaneous pain—were recorded in either group throughout the observation period. Furthermore, no instances of tooth discolouration were noted at any time point.

### 3.3. Results of Clinical Evaluation of Restorations

At each follow-up, restorations were qualitatively assessed for clinical performance using established criteria adapted from the modified USPHS system.

Throughout the 12-month observation period, all glass ionomer cement restorations showed stable clinical performance, with no cases of restoration loss or secondary caries recorded. Minor variations in marginal adaptation, surface texture or discolouration were noted, but no adverse trends or significant clinical failures occurred.

Due to the qualitative nature of the assessment and the limited sample size, formal scoring or statistical comparisons were not performed.

### 3.4. Pulp Vitality Testing Results

Pulp vitality in the experimental (A-PRF+) group was assessed at each follow-up using cold pulp testing (CPT) and electric pulp testing (EPT), with laser Doppler flowmetry (LDF) performed once per patient at either the 9- or 12-month recall.

Over the 12-month period, a progressive increase was observed in the number of teeth demonstrating positive responses to both cold and electric pulp tests. At the final recall, 71% of cases responded positively to CPT, 75% to EPT, and LDF confirmed pulp blood flow in 93% of cases.

Although EPT detected a slightly higher overall proportion of positive responses (n = 96) than CPT (n = 89), this difference was not statistically significant (*p* > 0.05). Detailed results for each time point are provided in Table 3.

### 3.5. Radiographic Outcomes

Radiographic evaluation revealed no cases of root fracture, internal and external root resorption, nor new periapical pathology in either the A-PRF+ or control (calcium hydroxide apexification) groups throughout the follow-up period. At baseline, periapical lesions were present in 12 cases per group. In the A-PRF+ group, 75% of lesions (n = 9) resolved by the 3-month recall, with complete healing observed in all cases by 9 months. In the control group, all lesions except one resolved by the end of therapy.

**Figure 1:**
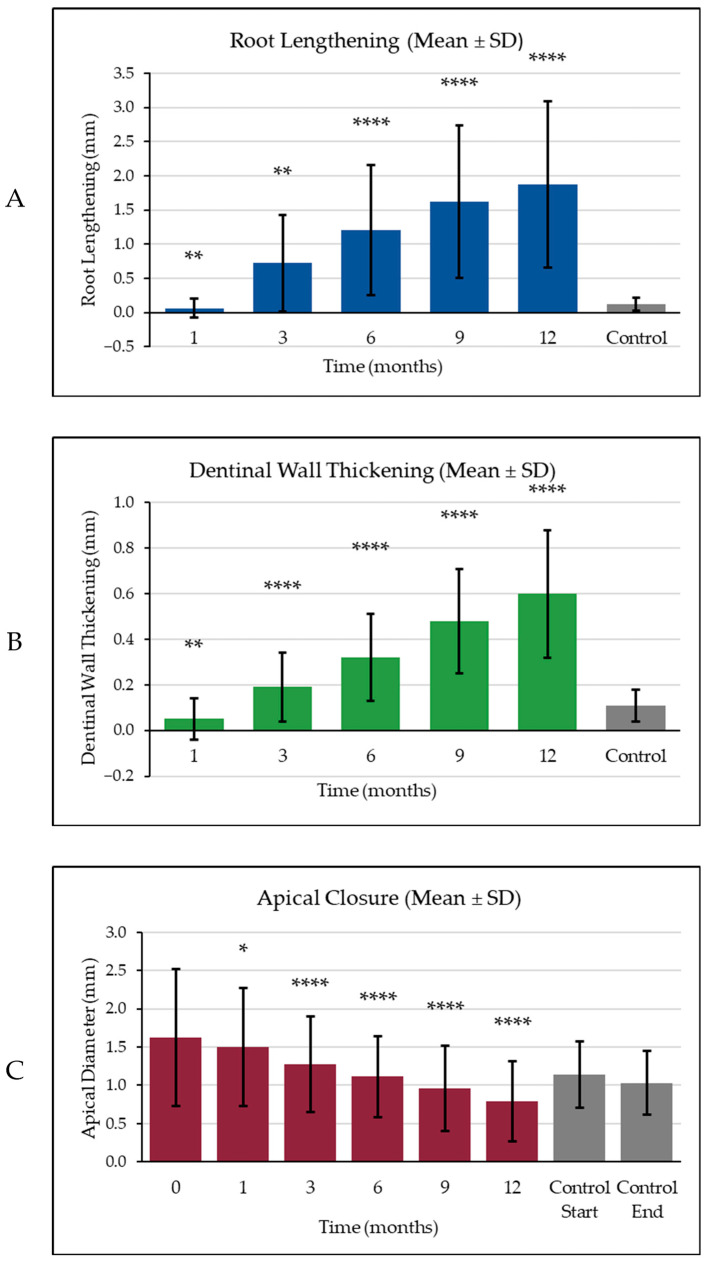
Comparative radiographic outcomes over 12 months for the A-PRF+ (coloured bars) and control (grey bars) groups: (**A**) root lengthening, (**B**) dentinal wall thickening and (**C**) apical closure, each presented as mean ± standard deviation (SD). Time points indicate months after baseline. Statistical significance (Mann–Whitney *U* test): *p* ≤ 0.05 (*), *p* ≤ 0.01 (**), *p* ≤ 0.0001 (****).

**Figure 2:**
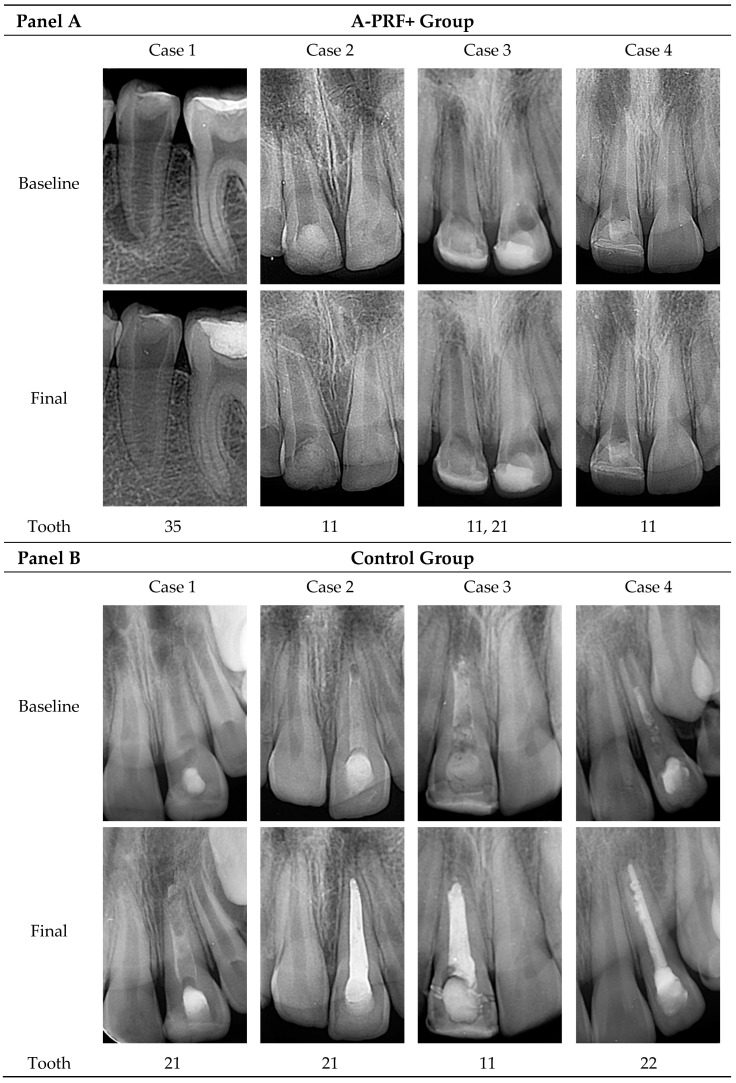
Representative periapical radiographs of immature necrotic permanent teeth treated with A-PRF+ protocol (Panel (**A**)) and calcium hydroxide apexification (Panel (**B**)). Each case shows baseline (upper row) and final (lower row) radiographs, with the designation of the treated tooth indicated below each case.

Significant and progressive improvements were observed in all radiographic parameters—root lengthening (RL), dentinal wall thickening (DWT) and apical closure (AC)—in the A-PRF+ group compared to the control, as shown in Table 4 and Figure 1. Representative radiographs illustrating these differences are presented in Figure 2. These findings demonstrate the enhanced regenerative capacity of A-PRF+ therapy relative to conventional apexification.

#### 3.5.1. Root Lengthening

Root lengthening in the A-PRF+ group showed continuous and progressive increases over the 12-month follow-up period. At 1 month, the mean increase was 0.06 ± 0.14 mm (25% of cases), rising to 0.72 ± 0.71 mm (75%) at 3 months, 1.21 ± 0.95 mm (89%) at 6 months, 1.62 ± 1.12 mm (93%) at 9 months and 1.88 ± 1.22 mm (93%) at 12 months.

In contrast, the control group demonstrated only minimal lengthening, with a mean increase of 0.12 ± 0.10 mm (68% of cases) by the end of therapy.

Between-group differences were statistically significant from early time points (*p* < 0.01) and became highly significant at 6, 9 and 12 months (*p* < 0.0001; Figure 1A).

#### 3.5.2. Dentinal Wall Thickening 

A-PRF+ treatment resulted in substantial gains in dentinal wall thickness, with mean increases from 0.05 ± 0.09 mm (32% of cases) at 1 month to 0.60 ± 0.28 mm (100%) at 12 months. By 6 months, most cases (96%) demonstrated measurable thickening (0.32 ± 0.19 mm), and all cases showed increases by 9 and 12 months.

In contrast, the control group exhibited only modest thickening, with a mean increase of 0.11 ± 0.07 mm (82% of cases) by the end of therapy.

Between-group differences were statistically significant at all time points and reached high significance (*p* < 0.0001) from 6 months onward (Figure 1B).

#### 3.5.3. Apical Closure

Progressive apical closure was observed in the A-PRF+ group, as indicated by a reduction in apical diameter from 0.12 ± 0.22 mm (32% of cases) at 1 month to 0.35 ± 0.38 mm (71%) at 3 months and reaching 0.84 ± 0.51 mm (100%) at 12 months.

The control group demonstrated only limited apical closure, with a mean reduction of 0.12 ± 0.09 mm (79% of cases) by the end of therapy.

Statistically significant differences between groups were evident from the earliest time point (*p* < 0.05 at 1 month) and became increasingly significant at subsequent follow-ups (*p* < 0.01 to *p* < 0.0001; Figure 1C).

#### 3.5.4. Radiographic Assessment of Apical Diameter and Treatment Outcomes

The distribution of apical diameter outcomes at baseline and final follow-up for the A-PRF+ and control groups, categorised by clinical suitability for subsequent conventional endodontic treatment, is summarised in Table 5. Outcome categories were defined as:

- Negative: apical diameter > 1.00 mm, not suitable for conventional endodontic treatment;
- Good: apical diameter 0.51–1.00 mm, adequate but potentially challenging;
- Very good: apical diameter 0.11–0.50 mm, favourable for treatment;
- Excellent: apical diameter ≤ 0.10 mm, ideal conditions for conventional endodontic treatment.

At baseline, the majority of cases in both the A-PRF+ (71.5%) and control (78.6%) groups had apical diameters ≥ 1.0 mm, indicating marked apical immaturity. Following treatment, the A-PRF+ group showed substantially greater apical closure, with 42.8% of cases (12/28) achieving an apical diameter ≤ 0.5 mm and 7.1% (2/28) attaining the excellent category (≤ 0.1 mm). In contrast, most cases in the control group (67.9%) remained in the good category (0.51–1.00 mm), with only one case (3.6%) classified as very good and none as excellent.

When stratified by clinical suitability for conventional endodontic treatment, the A-PRF+ protocol produced a notably higher proportion of very good and excellent outcomes compared to traditional apexification.

These findings demonstrate the superior efficacy of A-PRF+ revascularisation in achieving clinically meaningful apical closure, thereby enhancing the feasibility and predictability of subsequent conventional endodontic treatment relative to calcium hydroxide apexification.

### 3.6. Linear Regression Model for Predicting Apical Closure

Given the marked differences in rates of apical closure observed between groups, a linear regression model was developed to clarify the relationship between follow-up duration and reduction in apical diameter within the A-PRF+ cohort. The primary aim was to provide a quantitative tool for predicting the duration of A-PRF+ therapy required to achieve complete apical closure (apical diameter ≤ 0.1 mm), thereby supporting individualised treatment planning and prognosis.

The model was constructed using 157 observations from the experimental group. Apical closure (AC, in mm) was predicted based on treatment duration (MONTHS), patient age (AGE), sex (SEX; female or male) and aetiology (AETIOLOGY; caries or trauma) as follows:

(1) $$\mathrm{AC}\;=\;1.2101\;-\;0.0755\;\times \;\mathrm{MONTHS}\;+\;0.0273\;\times \;\mathrm{AGE}\;+\;0.1251\;\times \;\mathrm{SEX}{\_}_{F}\;-\;0.0154\;\times \;\mathrm{AETIOLOGY}{\_}_{C}$$

where

- AC: Predicted reduction in apical diameter (mm);
- MONTHS: Duration of A-PRF+ therapy (number of months);
- AGE: Patient age (years);
- SEX__F_: Sex (female = 1, male = 0);
- AETIOLOGY__C_: Aetiology (caries = 1, trauma = 0).

For example, to estimate apical closure after 6 months for a 12-year-old female with trauma (MONTHS = 6, AGE = 12, SEX__F_ = 1, AETIOLOGY__C_ = 0):

AC = 1.2101 − (0.0755 × 6) + (0.0273 × 12) + (0.1251 × 1) − (0.0154 × 0) = 1.2098

This suggests a predicted apical diameter reduction of approximately 1.21 mm after 6 months.

**Figure 3:**
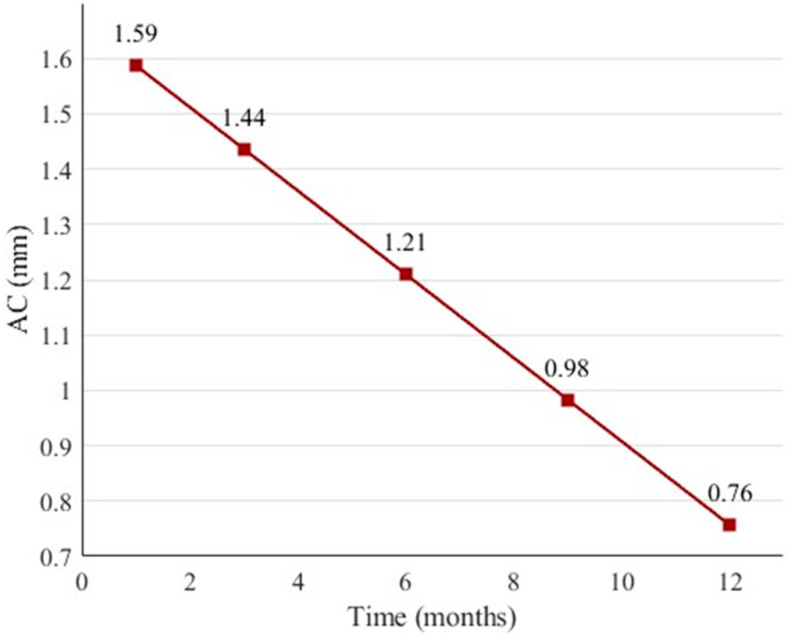
Scatter plot with regression line illustrating the reduction in apical diameter (AC, mm) as a function of treatment duration (months) in the A-PRF+ group. The line represents the fitted regression model, AC = 1.2101 − 0.0755 × MONTHS.

The regression plot is presented in Figure 3, illustrating the simplified relationship AC = 1.2101 − 0.0755 × MONTHS. Treatment duration was the only statistically significant predictor of apical closure, whereas age, sex and aetiology showed no significant effect (see Table 6). The full set of regression coefficients is presented in Table 6, and the overall model statistics are summarised in Table 7.

The model’s *F*-statistic of 10.2, with a corresponding *p* value of 2.49 × 10^−7^, indicates that the predictors collectively provide a significant improvement in predicting apical closure compared to a null model. Although treatment duration (MONTHS) was the only individually significant predictor (*p* < 0.0001), the combined inclusion of age, sex and aetiology enhanced the model’s explanatory power. The negative coefficient for MONTHS confirms that longer treatment duration is associated with a greater reduction in apical diameter.

While the model accounts for a moderate proportion of the variance (*R^2^* = 0.211), it offers a practical, evidence-based tool to assist clinicians in estimating expected outcomes following A-PRF+ regenerative therapy.

## 4. Discussion

This study provides compelling evidence for the clinical and radiographic efficacy of an advanced A-PRF+ revascularisation protocol compared with the established gold standard of calcium hydroxide apexification in the management of immature necrotic permanent teeth. Calcium hydroxide apexification was selected as the comparator due to its long-standing role as a reference treatment in this clinical context. The novel protocol assessed here—featuring the extended use of autologous A-PRF+ throughout the canal and pulp chamber, and exclusive glass ionomer cement for coronal sealing—was specifically designed to enhance procedural simplicity and biological compatibility in alignment with current tissue engineering principles [38,39,40].

### 4.1. Clinical Outcomes

The clinical outcomes with A-PRF+ were characterised by safety and excellent therapeutic effectiveness, with no adverse events or complications throughout follow-up. These results align with the primary aims of regenerative endodontic procedures (REPs): elimination of clinical symptoms and restoration of dental function [41]. Recent clinical trials and meta-analyses report comparable clinical success rates above 95% for various scaffolds used in REPs [16,17,18,42,43,44]. For example, a meta-analysis by Li et al. of 27 studies (676 immature permanent teeth) found success rates of 96.2% for asymptomatic cases and 95.6% overall in necrotic immature permanent teeth treated with REP protocols [16]. 

The absence of adverse events in the present study underscores the predictability and clinical relevance of the A-PRF+ protocol in the management of immature necrotic permanent teeth.

### 4.2. Radiographic Outcomes

The secondary aims of regenerative endodontic procedures—to encourage root maturation via root lengthening, dentinal wall thickening and apical closure [41]—were fully met with the A-PRF+ protocol. Statistically and clinically significant improvements were observed for all radiographic parameters when compared with the control group. At 12 months, the A-PRF+ group demonstrated extremely significant gains (*p* < 0.0001) in root lengthening (93%), dentinal wall thickening (100%) and apical closure (100%), whereas only modest changes were seen in the control group.

These findings not only surpass the majority of published outcomes for traditional apexification and other regenerative scaffolds, such as blood clot and standard PRF [19,42,45], but also align with the results of Qamar et al., who reported that A-PRF produced greater increases in root length and dentinal wall thickness than blood clot scaffolds, although not all differences reached statistical significance [19].

Notably, 42.8% of A-PRF+ cases achieved favourable or ideal apical diameters (≤ 0.50 mm) at final follow-up, compared to just 3.6% in the control group. Such apical narrowing is clinically significant as it supports predictable obturation and reduces the risk of reinfection or treatment failure. These results are in agreement with robust studies showing that A-PRF protocols promote significant apical closure, enhanced periapical healing and improved root development [42,46]. In addition, changes in apical closure and root maturation typically achieved after 8–9 months of traditional apexification were already evident in the A-PRF+ group within just one to three months, highlighting the accelerated regenerative effect of this approach. Similarly, Wakhloo et al. reported hard tissue barrier formation within three months in 90% of teeth treated with A-PRF [46].

The accelerated rate of root maturation and apical closure with A-PRF+ was also accompanied by rapid resolution of pre-existing periapical lesions: 75% of lesions resolved within three months, and complete healing was observed in all cases by nine months. This rapid recovery is most likely attributable to the well-documented angiogenic and tissue-regenerative effects of A-PRF+. Supporting this, Machut and Żółtowska, using recent three-dimensional radiographic analysis, found that A-PRF+ led to an 85.9% reduction in apical radiolucency volume at six months, compared with 72.3% for calcium hydroxide apexification, confirming the superior healing response provided by the A-PRF+ protocol [22].

Taken together, these results indicate that A-PRF+ as a scaffold in regenerative endodontic procedures can reliably achieve substantial root development and periapical healing at a rate and to an extent that exceed current standard therapies.

### 4.3. Pulp Vitality Test Outcomes

Pulp vitality testing in the A-PRF+ group demonstrated a clear, progressive increase in positive responses over the 12-month follow-up. By the final recall, 71% of treated teeth responded to cold pulp testing and 75% to electric pulp testing. These results are notably higher than those reported in a recent systematic review and meta-analysis, where pooled positive response rates in necrotic immature permanent teeth were 25.2% for electric pulp testing and just 14.5% for both cold and electric pulp tests combined [16].

This marked improvement is likely related to key features of the protocol, most importantly, the extension of the A-PRF+ scaffold into the coronal cavity. Placing the scaffold in the coronal region may promote a stronger pulpal response and improve the likelihood of detecting restored pulp vitality. In contrast, conventional regenerative endodontic procedures usually confine the scaffold to the apical region, which may lead to false-negative test results as the regenerated tissue is further from the site of stimulus. It is important to note that, according to histological studies, the tissues regenerated by these procedures are often cementum-like or bone-like rather than true pulp tissue, so positive sensibility test responses should be interpreted in this context [16].

Recognising the limitations of conventional sensibility tests—which do not directly measure pulpal blood flow and are particularly unreliable in immature or traumatised teeth [35]—pulp vitality assessment was supplemented by laser Doppler flowmetry. This technique directly measures pulpal blood flow and is considered a more sensitive and reliable method for evaluating pulp vitality, particularly in traumatised or developing teeth [35].

At the final recall, 93% of A-PRF+-treated teeth exhibited pulpal blood flow as measured by laser Doppler flowmetry. Taken together, these findings indicate that the described protocol achieves a high rate of functional pulp vitality, as evidenced by consistent results across multiple assessment modalities, and may offer significant advantages over conventional regenerative approaches for the management of immature necrotic permanent teeth.

### 4.4. Tooth Discolouration

The protocol adopted in this study incorporated several measures to minimise the risk of tooth discolouration and optimise aesthetic outcomes. Glass ionomer cement was selected as the sole coronal sealing material, while mineral trioxide aggregate (MTA, both white and grey forms) and Biodentine—materials commonly linked to tooth discolouration, particularly when in contact with blood or PRF scaffolds [9,47]—were intentionally excluded. As a result, no discolouration was observed in any of the treated teeth during the 12-month follow-up period.

Triple antibiotic paste (TAP), containing minocycline, a well-documented cause of crown staining, was carefully restricted to areas below the cementoenamel junction and prepared at a concentration of 5 mg/mL. This concentration was selected to provide adequate antibacterial action while staying below the threshold most frequently associated with clinically significant discolouration [48].

In addition, all residual TAP was meticulously removed from the pulp chamber prior to scaffold placement and final restoration, following best practices to minimise staining risk [49]. Together, these protocol modifications, combined with the use of glass ionomer cement as the coronal seal, enabled safe extension of the A-PRF+ scaffold into the coronal cavity without compromising aesthetics. This strategy is especially beneficial for teeth in the aesthetic zone, where preservation of natural tooth colour remains a critical clinical objective [49].

### 4.5. Linear Regression Model—Implications and Future Directions

To maximise the translational value of these findings, a preliminary linear regression model was constructed to predict apical closure over time in patients managed with the A-PRF+ protocol. The model identified treatment duration as the principal predictive variable, achieving overall statistical significance (*F*-statistic 10.2, *p* = 2.49 × 10^−7^) and accounting for approximately 21% of the observed variance in apical closure (*R^2^* = 0.211). While these results highlight the model’s potential as a clinical tool, they also underscore the necessity for further refinement.

It is anticipated that the addition of further patient, aetiology, tooth and treatment-specific variables—such as oral hygiene and periodontal status, time elapsed since the original trauma or onset of necrosis, preoperative presence and size of periapical lesion, location in the dental arch (maxillary versus mandibular, incisor versus premolar) and the type or brand of glass ionomer cement used for the coronal seal—may enhance predictive accuracy in future studies with larger sample sizes. At present, these variables are suggested based on clinical reasoning, and their significance should be confirmed in future investigations.

As an initial framework, the model provides a promising foundation for developing advanced, individualised predictive tools. Such tools could ultimately assist clinicians in optimising patient management, tailoring regenerative endodontic treatment and improving prognostic accuracy in routine practice.

### 4.6. Limitations and Future Research

Several limitations of the present study should be acknowledged, including its single-centre design, moderate sample size and non-randomised allocation of the control group. The relatively short follow-up period of 12 months may not capture long-term outcomes, late failures or changes in tooth vitality and function. Although radiographic assessments were conducted using a standardised and blinded protocol, evaluation remains subject to the inherent limitations of two-dimensional imaging, such as subjectivity and restricted resolution. In addition, the control group was selected retrospectively, which may introduce selection or information bias owing to differences in clinical management or incomplete historical records.

Furthermore, while pulp vitality was assessed using clinical sensibility tests and laser Doppler flowmetry, histological confirmation of true pulp regeneration was not possible within this clinical framework.

Future research should prioritise multicentre, randomised controlled trials with larger cohorts and extended follow-up to further validate these findings and identify additional prognostic indicators. Direct comparisons between this modified A-PRF+ protocol and established regenerative endodontic procedures will also be essential to determine whether the observed benefits can be consistently reproduced, both statistically and clinically.

## 5. Conclusions

The results of this study demonstrate that the advanced A-PRF+ protocol offers significant clinical and radiographic advantages in the management of immature necrotic permanent teeth. This protocol, which combines procedural simplicity, biological compatibility and robust regenerative efficacy, was associated with a high rate of clinical success, predictable root maturation, complete apical closure and a notable absence of adverse events, including tooth discolouration. Importantly, the protocol resulted in restoration of a continuous pulpal space without the formation of undesirable calcified barriers, re-establishment of the pulp chamber roof and consistent revascularisation evidenced by re-established pulpal blood flow, thereby confirming the primary study hypotheses.

Compared with both traditional calcium hydroxide apexification and conventional regenerative endodontic procedures, the A-PRF+ approach demonstrated superiority across multiple domains, including accelerated periapical healing, greater dentinal wall thickening and favourable clinical and aesthetic outcomes. These benefits were achieved using a streamlined, patient-centred protocol and further supported by preliminary modelling as a basis for future individualised predictive tools.

Although further multicentre, randomised studies with extended follow-up are required to confirm these results and refine prognostic models, the findings presented here position the A-PRF+ protocol as a promising foundation for broader clinical adoption and ongoing advancement in regenerative endodontic therapy.

## Figures and Tables

**Table 1:** Sample size calculations for root lengthening (RL), dentinal wall thickening (DWT) and apical closure (AC) based on preliminary data from the A-PRF+ and control groups. Parameters include means, standard deviations, mean differences, pooled standard deviations, effect sizes (Cohen’s *d*) and required sample sizes per group, adjusted for 20% loss to follow-up.

Outcome	Group	Mean (mm)	SD (mm)	Mean Difference	Pooled SD	Effect Size (Cohen’s *d*)	Required Size ^1^	Adjusted Size ^2^
RL	A-PRF+	1.06	1.15	0.97	0.83	1.19	20	25
	Control	0.09	0.11					
DWT	A-PRF+	0.33	0.20	0.25	0.15	1.68	11	14
	Control	0.08	0.06					
AC	A-PRF+	0.68	0.46	0.57	0.32	1.73	10	13
	Control	0.11	0.09					

^1^ Calculated for 95% statistical power, *α* = 0.05, two-sided test. ^2^ Adjusted size allows for 20% loss to follow-up.

**Table 2:** Baseline demographic and clinical characteristics of the A-PRF+ and control groups. Data are presented as mean ± SD for continuous variables, or as n (%) for categorical variables.

Variable	A-PRF+ Group (n = 28)	Control Group (n = 28)
Age, years (mean ± SD)	11.36 ± 2.39	11.57 ± 2.08
Age range, years	8–17	8–15
Male, n (%)	13 (46.4)	19 (67.9)
Female, n (%)	15 (53.6)	9 (32.1)
Caries, n (%)	9 (32.1)	13 (46.4)
Trauma, n (%)	19 (67.9)	15 (53.6)
Maxillary central incisor, n (%)	24 (85.7)	25 (89.3)
Maxillary lateral incisor, n (%)	1 (3.6)	3 (10.7)
Mandibular 2nd premolar, n (%)	3 (10.7)	0 (0)
Cvek stage 1/2/3/4, n (%) *	8/5/7/8 (28.6/17.9/25/28.6)	1/5/11/11 (3.6/17.9/39.3/39.3)
Periapical lesion, n (%)	12 (42.9)	12 (42.9)
Therapy duration, months	—	8.64 ± 2.78

* Cvek stages: 1, apical diameter > 2 mm; 2, apical diameter 1.5–2 mm; 3, apical diameter 1–1.49 mm; 4, apical diameter 0.6–0.99 mm.

**Table 3:** Pulp vitality assessment results over 12 months in the A-PRF+ group, using cold pulp testing (CPT), electric pulp testing (EPT) and laser Doppler flowmetry (LDF). Data are presented as the percentage of positive responses (number of positive responses/total cases).

Time (Months)	CPT % (n/28)	EPT % (n/28)	LDF % (n/28)
1	43% (12/28)	54% (15/28)	—
3	61% (17/28)	68% (19/28)	—
6	71% (20/28)	71% (20/28)	—
9	71% (20/28)	75% (21/28)	93% (26/28) *
12	71% (20/28)	75% (21/28)	93% (26/28) *

* LDF was performed once per patient at either the 9- or 12-month recall. “—” indicates that the test was not performed at this time point.

**Table 4:** Quantitative radiographic outcomes for root lengthening (RL), dentinal wall thickening (DWT) and apical closure (AC) in the A-PRF+ and control groups at designated time points. Data are presented as means ± SDs (mm), n (%) positive cases and between-group *p* values (Mann–Whitney *U* test). Statistical significance: *p* ≤ 0.05 (*)*, p* ≤ 0.01 (**), *p* ≤ 0.0001 (****).

Outcome	Group	Time (Months)	Mean ± SD (mm)	n/28 (%)	*p* Value
RL	A-PRF+	1	0.06 ± 0.14	7/28 (25%)	0.0019 **
		3	0.72 ± 0.71	21/28 (75%)	0.0011 **
		6	1.21 ± 0.95	25/28 (89%)	<0.0001 ****
		9	1.62 ± 1.12	26/28 (93%)	<0.0001 ****
		12	1.88 ± 1.22	26/28 (93%)	<0.0001 ****
	Control	Final	0.12 ± 0.10	19/28 (68%)	
DWT	A-PRF+	1	0.05 ± 0.09	9/28 (32%)	0.0011 **
		3	0.19 ± 0.15	23/28 (82%)	<0.0001 ****
		6	0.32 ± 0.19	27/28 (96%)	<0.0001 ****
		9	0.48 ± 0.23	28/28 (100%)	<0.0001 ****
		12	0.60 ± 0.28	28/28 (100%)	<0.0001 ****
	Control	Final	0.11 ± 0.07	23/28 (82%)	
AC	A-PRF+	1	0.12 ± 0.22	9/28 (32%)	0.037 *
		3	0.35 ± 0.38	20/28 (71%)	<0.0001 ****
		6	0.51 ± 0.43	27/28 (96%)	<0.0001 ****
		9	0.67 ± 0.44	28/28 (100%)	<0.0001 ****
		12	0.84 ± 0.51	28/28 (100%)	<0.0001 ****
	Control	Final	0.12 ± 0.09	22/28 (79%)	

**Table 5:** Distribution of apical diameter categories at baseline and final follow-up in the A-PRF+ and control groups, classified by clinical suitability for conventional endodontic treatment.

Assessment	Apical Diameter (mm)	A-PRF+ Group n (%)	Control Group n (%)	Outcome Category
Baseline	>2.00	8 (28.6%)	1 (3.6%)	
	1.50–2.00	5 (17.9%)	5 (17.9%)	
	1.00–1.49	7 (25.0%)	11 (39.3%)	
	0.60–0.99	8 (28.6%)	11 (39.3%)	
Final	>1.00	8 (28.6%)	8 (28.6%)	Negative ^1^
	0.51–1.00	8 (28.6%)	19 (67.9%)	Good ^2^
	0.11–0.50	10 (35.7%)	1 (3.6%)	Very good ^3^
	≤0.10	2 (7.1%)	0 (0%)	Excellent ^4^

^1^ Negative—apical diameter > 1.00 mm; unsuitable for conventional endodontic treatment. ^2^ Good—apical diameter 0.51–1.00 mm; adequate for conventional endodontic treatment. ^3^ Very good—apical diameter 0.11–0.50 mm; favourable for conventional endodontic treatment. ^4^ Excellent—apical diameter ≤ 0.10 mm; ideal for conventional endodontic treatment.

**Table 6:** Linear regression model coefficients for predicting apical closure in the A-PRF+ group.

Predictor	Estimate	SE	*t* Statistic	*p* Value
Intercept	1.2101	0.281	4.31	<0.001
MONTHS	−0.0755	0.012	−6.26	<0.0001
AGE	0.0273	0.023	1.21	0.229
SEX__F_	0.1251	0.106	1.18	0.238
AETIOLOGY__C_	−0.0154	0.111	−0.14	0.890

**Table 7:** Key statistical parameters from the regression analysis.

Statistic	Value
Number of observations	157
Error degrees of freedom	152
*R*-squared	0.211
Adjusted *R*-squared	0.191
Root mean squared error	0.649
*F*-statistic vs. constant model	10.2
*F*-statistic *p* value	2.49 × 10^−7^

## Data Availability

The original contributions presented in this study are included in the article. Further inquiries can be directed to the corresponding author.

## Abbreviations

The following abbreviations are used in this manuscript:

AAE	American Association of Endodontists
A-PRF	Advanced Platelet-Rich Fibrin
A-PRF+	Advanced Platelet-Rich Fibrin Plus
AC	Apical Closure
ANOVA	Analysis of Variance
CEJ	Cementoenamel Junction
CPT	Cold Pulp Testing
DWT	Dentinal Wall Thickening
EPT	Electric Pulp Testing
ESE	European Society of Endodontology
GIC	Glass Ionomer Cement
GICs	Glass Ionomer Cements
L-PRF	Leukocyte- and Platelet-Rich Fibrin
LDF	Laser Doppler Flowmetry
MTA	Mineral Trioxide Aggregate
PPP	Platelet-Poor Plasma
PRF	Platelet-Rich Fibrin
REP	Regenerative Endodontic Procedure
REPs	Regenerative Endodontic Procedures
RL	Root Lengthening
ROI	Region of Interest
S-PRF	Standard Platelet-Rich Fibrin
SD	Standard Deviation
TAP	Triple Antibiotic Paste
USPHS	United States Public Health Service
